# Short-term detraining in semi-professional male football players: impact of a 5-day mid-season break on performance - an observational study

**DOI:** 10.1186/s13102-026-01654-4

**Published:** 2026-04-09

**Authors:** João G. Saldanha, Francisco Santos, Diogo V. Martinho, Hugo Sarmento, Adilson Marques, Pedro Campos, Krzysztof Przednowek, Élvio R. Gouveia

**Affiliations:** 1 Department of Physical Education and Sport, University of Madeira, Funchal, 9020-105 Portugal; 2 LARSYS, Interactive Technologies Institute, Funchal, 9020-105 Portugal; 3 Universidade de Coimbra, CIPER, Faculdade de Ciências do Desporto e Educação Física, Coimbra, Portugal; 4 CIPER, Faculdade de Motricidade Humana, Universidade de Lisboa, Cruz-Quebrada-Dafundo, Portugal; 5 ISAMB, Faculdade de Medicina, Universidade de Lisboa, Lisboa, Portugal; 6 Department of Informatics Engineering and Interactive Media Design, University of Madeira, Funchal, Portugal; 7 Wowsystems Informática Lda, Funchal, Portugal; 8 Faculty of Physical Culture Sciences, Medical College, University of Rzeszów, Rzeszów, Poland; 9 Swiss Center of Expertise in Life Course Research LIVES, Carouge, 1227 Switzerland

**Keywords:** detraining, physical fitness, performance, football players, repeated sprint ability, in-season break

## Abstract

**Background:**

Detraining is a partial reduction or total stoppage of training workloads, resulting in physical and physiological modifications. Prior studies have shown significant reductions in strength and power typically after long-term interruptions (i.e., > 4 weeks), whereas evidence for short-term detraining (i.e., < 4 weeks) remains inconclusive. This observational study describes acute changes in the physical abilities of football players associated with a 5-day in-season break under real-world conditions.

**Methods:**

In this uncontrolled observational study, repeated sprinting ability (primary outcome), body composition, vertical jump performance, and maximum thigh adduction and abduction strength were assessed in 17–21 semi-professional male football players (*n* = 17 for the primary outcome; other tests ranged from 17 to 21) before and after a 5-day in-season break (age: 21.5 ± 1.37 years; height: 178.96 ± 6.59 cm). Descriptive statistics and paired t-tests were used for comparisons. Intra-individual differences were also calculated.

**Results:**

After the 5-day break, repeated sprinting ability was significantly worse. Total time to complete the RAST was approximately 1.0 s higher (before: 29.89 ± 1.14 s; after: 30.77 ± 1.62 s, *p* = 0.009, d = 0.634). Peak power and maximal velocity showed mean reductions of 55 watts (before: 892.34 ± 125.46 W; after: 837.54 ± 108.92 W, *p* = 0.049, d = -0.427) and 0.65 km.hˉ¹ (before: 26.86 ± 1.03 km.hˉ¹; after: 26.21 ± 1.35 km.hˉ¹, *p* = 0.030, d = -0.489), respectively, indicating small-to-moderate effect sizes. Vertical jump performance showed slight, non-significant improvements. No significant changes were found for maximum thigh strength or body composition.

**Conclusion:**

A brief 5-day in-season break under uncontrolled, real-world conditions was associated with small-to-moderate impairments in repeated sprinting ability, while body composition, maximal strength, and vertical jump performance were largely maintained. These findings underscore the need for practitioners to prioritise targeted interventions for repeated sprinting ability following short in-season breaks, alongside strategies to restore performance without compromising technical-tactical demands.

**Supplementary Information:**

The online version contains supplementary material available at 10.1186/s13102-026-01654-4.

## Background

Detraining can be defined as a partial reduction or total interruption of training loads, leading to a series of physical and physiological changes [1–3]. Depending on the duration, shorter or longer than 4 weeks, it can be classified as either short or extended [4]. In football, detraining can occur after off-season periods, mid-season breaks or injury-related absences [5].

In order to achieve high levels of performance in football, it is imperative to develop the technical, tactical, physical and psychological components [6]. In the pre-season, training focuses on enhancing physical fitness, whereas during the in-season phase, training emphasises refining game strategies and optimising performance while sustaining physical fitness levels [7]. Physical fitness plays an essential role due to its positive anatomical, physiological, functional and biomechanical effects [8, 9]. Football is an activity that involves intermittent, high-intensity, acyclic efforts and requires a large volume of varied motor actions, in which aerobic and anaerobic fitness, strength, speed, agility, flexibility and power are decisive for performance [10, 11]. The football match is characterised by moments of high intensity and short duration, with brief recovery intervals, causing an interplay between the predominance of aerobic and anaerobic mechanisms [12]. The aerobic system is used in approximately 88% of the football match, while the other 12% is devoted to the high-intensity, short-duration anaerobic system [8, 10, 13]. Anaerobic capacity and power are important for players to tolerate repeated high-intensity stimuli, such as accelerations, decelerations and changes of direction.

In contemporary football practice, recovery and readiness are typically monitored through a combination of subjective and objective indicators, including self-reported well-being and perceived exertion scales, as well as internal and external load measures such as heart rate-based indices, total distance, high-speed running, number of sprints, accelerations, and others, which are used to infer fatigue and manage training load [14–16]. Alongside these methods, performance-based field tests are widely employed to characterise physical fitness and neuromuscular status, with clubs routinely assessing body composition, lower-limb strength, vertical jump performance and, in some cases, repeated sprinting ability, as these measures are closely related to match demands and are sensitive to training-induced changes in performance and fatigue [17, 18]. In this context, the present study focuses on repeated sprinting ability, vertical jump height, maximal thigh adduction and abduction strength, and body composition as practical, field-based outcomes that reflect key aspects of players’ physical readiness around a short in-season break under real-world conditions.

### Related work

High-intensity training is a more powerful stimulus for skeletal muscle adaptation than moderate-intensity training [7, 19]. High-intensity interval training (HIIT) is widely used during retraining periods for its effectiveness in enhancing aerobic fitness, repeated sprinting ability, and sprint performance [20]. Similarly, small-sided games (SSGs) are frequently employed due to their intense physiological demands and their alignment with match-play work rates [21], targeting aerobic power and integrating sport-specific skills within game-like scenarios, which makes them a valuable tool for athlete retraining [22]. The magnitude of changes observed after detraining can vary depending on the fitness level and the duration of training cessation [4, 23]. Previous studies [4, 23] have shown significant reductions in strength and power performance in athletes with different training backgroundsafter long-term interruptions (i.e., periods longer than 4 weeks). The off-season of competitive football schedules typically involves a training cessation lasting 4 to 6 weeks [24]. This type of prolonged reduction or hiatus in training can impair body composition and physical fitness, with studies in football players showing increases in fat mass and declines in aerobic fitness, strength, power, speed, and change-of-direction abilities [25, 26]. In particular, Clemente et al. (2022) [22] observed that a 4-week detraining period in youth male soccer players was associated with impairments in fat mass, aerobic fitness, vertical and horizontal jump performance, linear sprinting, change of direction and balance, highlighting the potential breadth of long-term detraining effects.

By contrast, evidence regarding short-term detraining (i.e. < 4 weeks) remains inconclusive and, at times, contradictory [26–28]. In a research study conducted with footballers from a university team, after a 1-week stoppage in training, performance in the total time of repeated sprints (45.7 ± 2.6 s vs. 48.0 ± 2.6 s, *p* = 0.01) and the fatigue index (5.8% ± 2.8% vs. 7.8% ± 3.2%, *p* = 0.04) showed a decline [29]. Additionally, 2 weeks of rest resulted in significant declines in intermittent cardiorespiratory fitness and repeated sprinting capacity in semi-professional footballers [7]. However, Rodríguez-Fernández et al. (2018) [30] found no significant changes in intermittent endurance in young professional and elite footballers for the same two-week break duration. Other investigations, involving interruptions of 3.5 to 28 days, have also reported no meaningful decrements in strength, speed or power in professional under-20 footballers and athletes from other sports [27, 31–33], whereas several studies have suggested decreases in physical conditioning after short-term detraining in elite football players [34, 35]. The underlying reasons for these contrasting results are unclear and may be due to differences in sports and testing methods [7].

Short-term interruptions are common within the competitive season, including brief mid-season breaks or short gaps between fixtures. However, there is limited evidence on how very short in-season breaks (≤ 7 days) affect key physical qualities in semi-professional or professional footballers, particularly under uncontrolled, real-world conditions. A deeper understanding of this issue is crucial for practitioners, as it will help to: (1) realise which weaknesses are most likely to emerge after the break; and (2) prioritise which physical skills to work on immediately after resumption, since opportunities for physical and technical development are often limited [26, 27]. From an ecological perspective, in-season breaks of approximately one week are representative of scheduling patterns in major European leagues [36, 37], and the team examined in the present study experienced a competitive interval of around three weeks across the Christmas period, within which a 5-day complete training cessation occurred. Thus, there is a need for applied, observational research examining how very short in-season breaks influence physical performance-related capacities in real-world football settings.

Therefore, this study aimed to analyse the acute effect of a 5-day in-season break on the physical qualities of semi-professional football players, particularly in terms of body composition and short-term maximal efforts. More specifically, we sought to examine changes in repeated sprinting ability (primary outcome), as well as in body composition, maximal thigh adduction and abduction strength, and vertical jump performance (secondary outcomes). Evidence suggests that certain capacities may be particularly vulnerable to short-term detraining, especially those dependent on high-intensity anaerobic mechanisms. We hypothesised that a 5-day in-season detraining period would be associated with impaired repeated sprinting ability, with minimal changes in maximal strength and body composition, and negligible or small changes (including potential minor improvements) in vertical jump performance. Hence, the study places special emphasis on repeated sprinting ability as the key performance marker.

## Methods

This study employed an observational, uncontrolled pre–post design conducted under real‑world mid‑season conditions in a semi‑professional football team.

### Ethics

The current project was conducted according to the Declaration of Helsinki and was approved by the Ethical Committee from the University of Lisbon Faculty of Human Kinetics (CEIFMH, No. 34/2021). This study was conducted following the Declaration of Helsinki, and informed written consent was obtained from all participants after a detailed description of the study procedures. Data were anonymised by assigning a specific code to each player. The participants were recruited on December 21, 2023, and data collection was completed by December 29, 2023.

### Participants

**Fig. 1:**
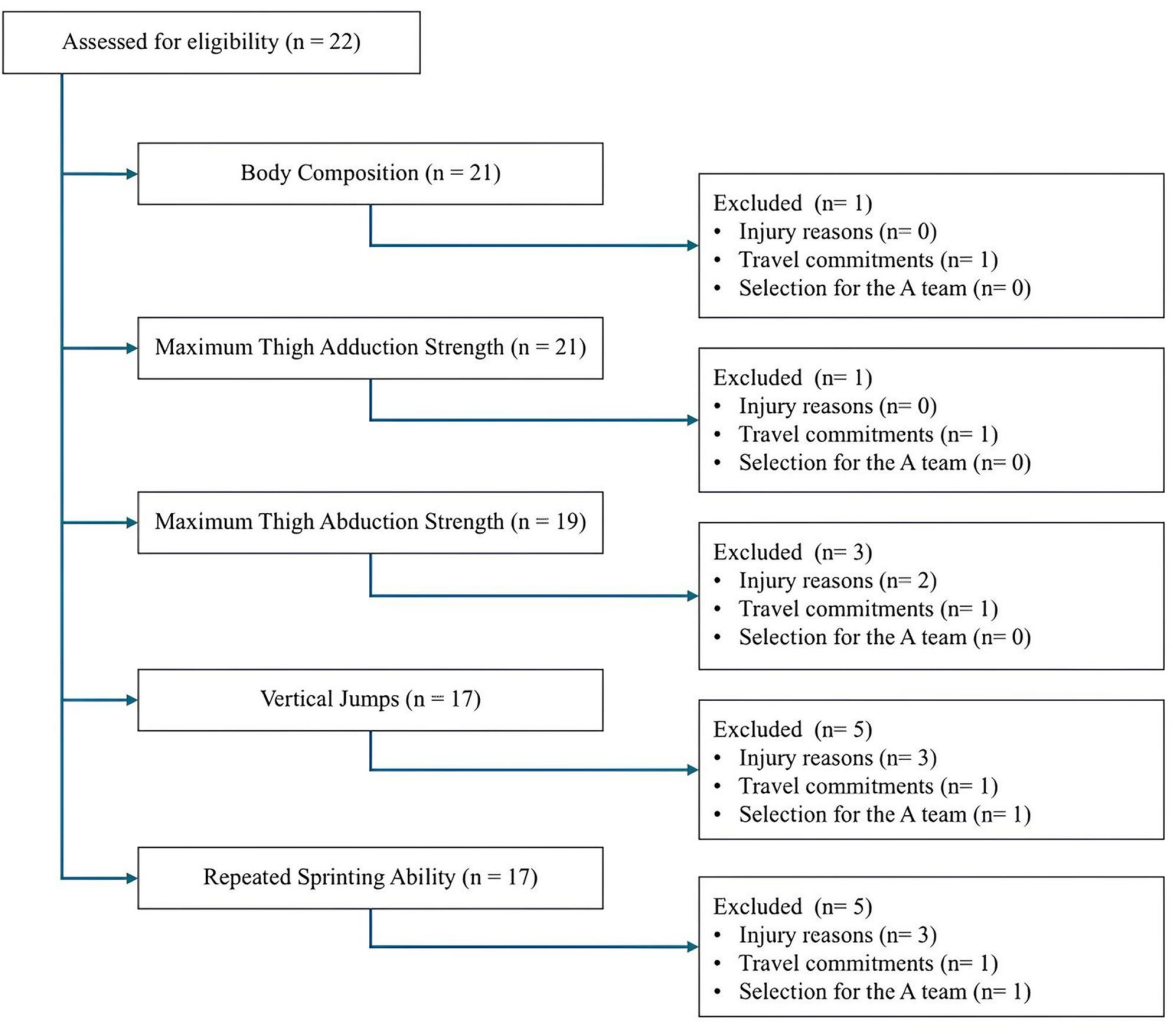
CONSORT-style flow diagram of participant inclusion and exclusion per assessment test

The final analysed sample for the primary outcome (repeated sprinting ability) consisted of 17 players (*n* = 17), drawn from an initial eligible sample of 22 semi-professional footballers. Due to the real-world constraints of a professional football environment, the final sample size varied between tests, from 17 to 21 individuals (age: 21.5 ± 1.37 years; height: 178.96 ± 6.59 cm; body mass: before the interruption = 75.08 ± 8.45 kg, after five days of detraining = 75.36 ± 8.33 kg), as shown in Fig. 1. Variability in the sample primarily resulted from injuries or progressive return-to-play protocols, which restricted certain individuals from maximal running, jumping, or strength tests, depending on the nature of their injury. Additionally, player absences due to travel commitments or selection for the A team contributed to incomplete participation in some assessments. For each variable, only participants with complete pre- and post-detraining data were included in the analyses, resulting in test-specific sample sizes. This pattern of missingness, likely not completely at random, may limit the generalisability of findings for individual tests. No a priori power calculation was performed, as the sample was determined by the fixed size of a single team squad. Accordingly, the analyses should be regarded as preliminary and exploratory in nature.

Players were part of a team competing in the 4th Portuguese Division. Although this league is classified as semi-professional, all players were full-time club professionals who belonged to a B team. These players adhered to a training regimen equivalent to that of professional teams, consisting of 5 to 6 training sessions per week, in addition to weekly competitive matches. Moreover, some individuals were periodically selected to train and/or compete in the club’s A team, which participated in the 2nd professional league. During the pre- and post-detraining assessments, only one player was excluded from certain tests due to A team call-up, as shown in Fig. 1.

The inclusion criteria were players from the team squad who were not currently injured and from all positions on the pitch, including goalkeepers. The exclusion criteria were: (1) players who took part in only one of the assessments (pre- or post-training); (2) individuals who, for reasons of injury and/or contraindication from the medical department, were prevented from taking part in a specific test. The participants’ flow through the different tests is illustrated in Fig. 1, providing in-depth information on inclusions and exclusions by assessment. The final sample sizes for each variable analysed are then summarised in Fig. 1.

### Study design

The detraining period took five days and occurred in the mid-season, specifically during the Christmas break, as part of the team’s usual competitive schedule rather than as an experimental interruption imposed. During this phase, the players were not involved in any training sessions or matches. Although their physical activity was not controlled, the subjects were advised to refrain from self-administered vigorous physical exercise. Nutritional dietary and fluid intake, and sleep were not monitored during the break.

**Fig. 2:**
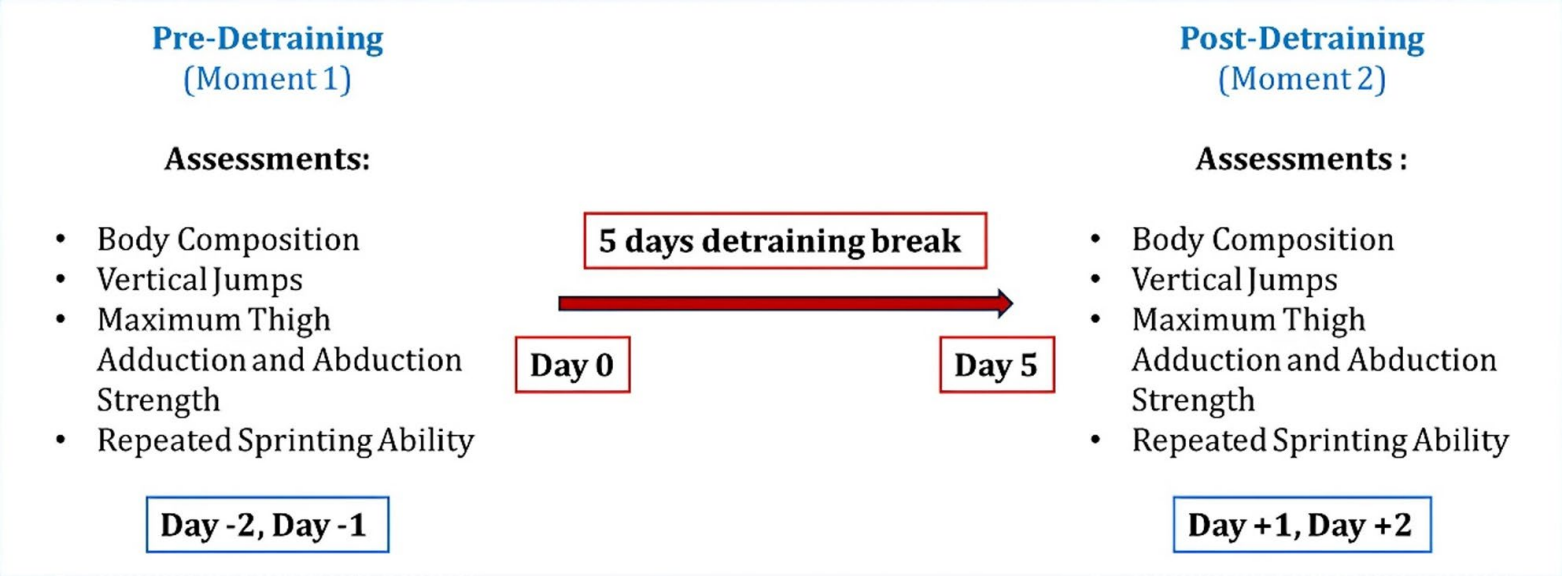
A schematic illustration of the experimental design

For logistical reasons, players were scheduled across two assessment days before the stoppage (Day − 2 and Day − 1) and two assessment days after the stoppage (Day + 1 and Day + 2), according to their availability. Players underwent the testing battery once in the pre‑detraining period (Day − 2/Day − 1 testing window) and once in the post‑detraining period (Day + 1/Day + 2 testing window), ensuring each test protocol was performed only once pre- and once post-detraining. Pre- and post-detraining evaluations were scheduled at the same time of day to minimise potential circadian influences on performance. The participants were assessed in terms of their body composition, followed by vertical jumps, maximum thigh adduction and abduction strength and, finally, they underwent a field test to assess their ability to sprint repeatedly. Because testing could only be conducted in a restricted time window before training and staff and equipment were limited, the day on which each player was assessed (Day − 2 vs. Day − 1, and Day + 1 vs. Day + 2) was determined by practical constraints (e.g. travel plans, authorised absences, A‑team commitments), and it was not always possible to retest each player on the same relative day at both time points. This scheduling feature represents an additional potential source of variability in the pre–post comparisons and reflects the ecological nature of data collection in this real‑world setting. The procedures were identical for both the first (pre-detraining) and second (post-detraining) assessment periods. Players were not informed about the specific study hypothesis or that the two testing sessions would be compared. They believed both assessments were routine club monitoring evaluations, similar to those typically conducted at the start of each season, which helped minimise potential bias in their performance. The assessors followed standardised instructions and protocols in both moments to ensure consistency in data collection. A schematic illustration of the experimental design is shown in Fig. 2.

### Measures

#### Maximum thigh adduction and abduction strength

The maximum isometric strength of the thigh adductors and abductors was estimated using a portable dynamometer (Smart Groin Trainer, Neuro Excellence, Portugal). The assessment protocol was defined taking into account some reference studies [38–42], following the sequence outlined below: (1) Place the subject in dorsal decubitus on the mat, with a 45º flexion at the hip joint level and knees flexed to 90º; (2) Place the dynamometer between the subject’s knees (medial femoral condyle area); (3) In the case of the 1st assessment, the subject should be given 2 attempts to familiarise them with the test; (4) Perform two repetitions of maximum force for 7 s - for both the adduction and abduction tests; (5) Recovery time between repetitions: 30 s. The maximum force values achieved during the first 7 s of contraction, in adduction and abduction, were subsequently retained for analysis. According to the measurement units provided by the device, hip strength was expressed in kilogram-force (kg.f) corresponding to the force exerted by a 1 kg mass under standard gravity (1 kg.f ≈ 9.81 N).

Previous studies investigating the intra-rater reliability of adduction and abduction hip strength assessments, in adult male football players [43] and runners [44], using hand held dynamometers, revealed good to excellent consistency, with intraclass correlation (ICC) values between 0.76 and 0.98 and measurement errors (SEM) between 0.58 and 17.2 N, thus endorsing the reproducibility, accuracy and reliability of these tests in senior male footballers.

#### Vertical jumps

The Optojump Next analysis and measurement system (Microgate, Bolzano, Italy) was used to assess vertical jumping ability, considering two types of jump: Countermovement Jump (CMJ) and Squat Jump (SJ).

In the case of the CMJ, this involves a long-duration eccentric-concentric action (contact time > 200–250 ms), in which the participants adopt an initial position with the Lower Limbs (LL) at 180º. In the eccentric phase, the LL are brought up to around 90º. The hands were always placed on the waist and the torso was upright, with the feet apart. The depth of the countermovement was self-regulated by the participants, although they were instructed to squat down to around 90º of knee flexion. In the flight phase, they were forced to maintain LL extension [45].

Regarding the SJ, the starting position is 90º knee flexion, a static position (2–3 s), always with the hands on the waist and the torso upright, followed by a vertical jump without countermovement and reception with the front of the foot and the knees in extension.

Participants had been previously familiarised with the assessment protocols and were encouraged to perform three maximum effort vertical jumps to the CMJ and SJ, with their hands on their waists. If the execution of the movement was considered incorrect, the jump was repeated. Regarding rest time between jumps, there is no consensus in the literature, with studies adopting an effort: rest duration ratio of 1:6 [46]; rest times of 20 [47] and 30 s [48, 49], and even others recommending up to 1 min [50]. Thus, to ensure the minimum recovery time between jumps, bearing in mind the range of variation in this matter, as well as to fulfil the time available for all the assessments, a 30-second recovery period was established between jumps and repetitions, which is a sufficient period to guarantee the validity of the results and has been applied in recent research [46–49]. Some studies also demonstrated very high test-retest reliability for the CMJ and SJ, with ICC values above 0.9 and coefficient of variation (CV) percentages below 8.5%, indicating excellent consistency of measurements over time [51, 52]. Using the specific software for this instrument (Optojump Next software, version 11324), the maximum height values for both jumps were collected for analysis.

#### Repeated sprinting ability – “Running Anaerobic Sprint Test” (RAST)

Anaerobic performance (Repeated Sprinting Ability) was assessed using a protocol called RAST, which involved 6 maximal efforts of 35 m, with 10 s of active recovery between each sprint. The measuring instrument used was an infrared photocell system (Witty, Micro Gate System, Mahopac, NY, USA). Repeated sprinting ability was considered the primary outcome of the study and was assessed through RAST total time, peak power, and maximum speed.

Before the test, the players performed a warm-up of between 10 and 15 min, which included running, dynamic stretching and acceleration. After a brief passive rest, the athletes began the RAST protocol. No separate familiarisation trials were conducted, as all subjects had extensive prior experience with the test. Additionally, due to the exhaustive nature of the test, conducting separate familiarisation trials was avoided to prevent undue fatigue and to guarantee physical readiness for the actual testing sessions. Nevertheless, we recognise this as a minor limitation, as day-to-day performance variability may still occur even in experienced athletes.

The subjects were instructed to run the 35 m distance as fast as possible, to decelerate after the finish line and, before the end of the 10 s active recovery period, to return to the starting line for the next repetition. This procedure was repeated until the 6 sprints were completed. Using the software specific to photocells (WittyManager, version 1534), it was possible to obtain information on the times of each of the sprints completed. From these times, it was possible to assess the maximum speed reached and the total time of the 6 × 35 m sprints [53]. Peak/maximum, minimum and average power, relative peak power and the fatigue index (FI) were also analysed.

i. $$\mathrm{Power}\;=\;\mathrm{Body}\;\mathrm{Mass}\times\;\mathrm{Distance}^{2}\;\div\;\mathrm{Time}^{3}$$
ii. $$\mathrm{Peak}\;\mathrm{power}\;\left(\mathrm{PP}\right)\;-\;\mathrm{highest}\;\mathrm{power}\;\mathrm{value}$$
iii. $$\mathrm{Relative}\;\mathrm{Peak}\;\mathrm{Power}\;\left(\mathrm{RPP}\right)\;=\;\mathrm{PP}\div\;\mathrm{Body}\;\mathrm{Mass}$$  
iv. $$\begin{aligned}\mathrm{Fatigue}\;\mathrm{Index}\;\left(\mathrm{FI}\right)\;&=\;\left(\mathrm{maximum}\;\mathrm{power}\;-\;\mathrm{minimum}\;\mathrm{power}\right)\\\;&\div\;\mathrm{total}\;\mathrm{time}\;\mathrm{of}\;6\;\mathrm{sprints}\end{aligned}$$  
v. $$\mathrm{Maximum}\;\mathrm{Speed}\;=\;\mathrm{Distance}\div\;\mathrm{Best}\;\mathrm{Time}\;\mathrm{of}\;\mathrm{the}\;6\;\mathrm{sprints}$$  

The environmental settings were the same for both pre- and post-detraining assessments, with the test being performed on a dry artificial grass surface. In footballers, RAST has shown high reproducibility for peak power (ICC = 0.88; CV = 10.2%) and mean power (ICC = 0.96; CV = 5.9%), when performed on grass with football boots, just as on rigid surfaces, indicating low measurement error and strong test-retest reliability [54]. However, the reliability of the FI was lower, with a higher CV (14.4%) and typical error (5.7%), suggesting greater measurement error and reduced reproducibility for this metric [54].

### Statistical analysis

**Fig. 3:**
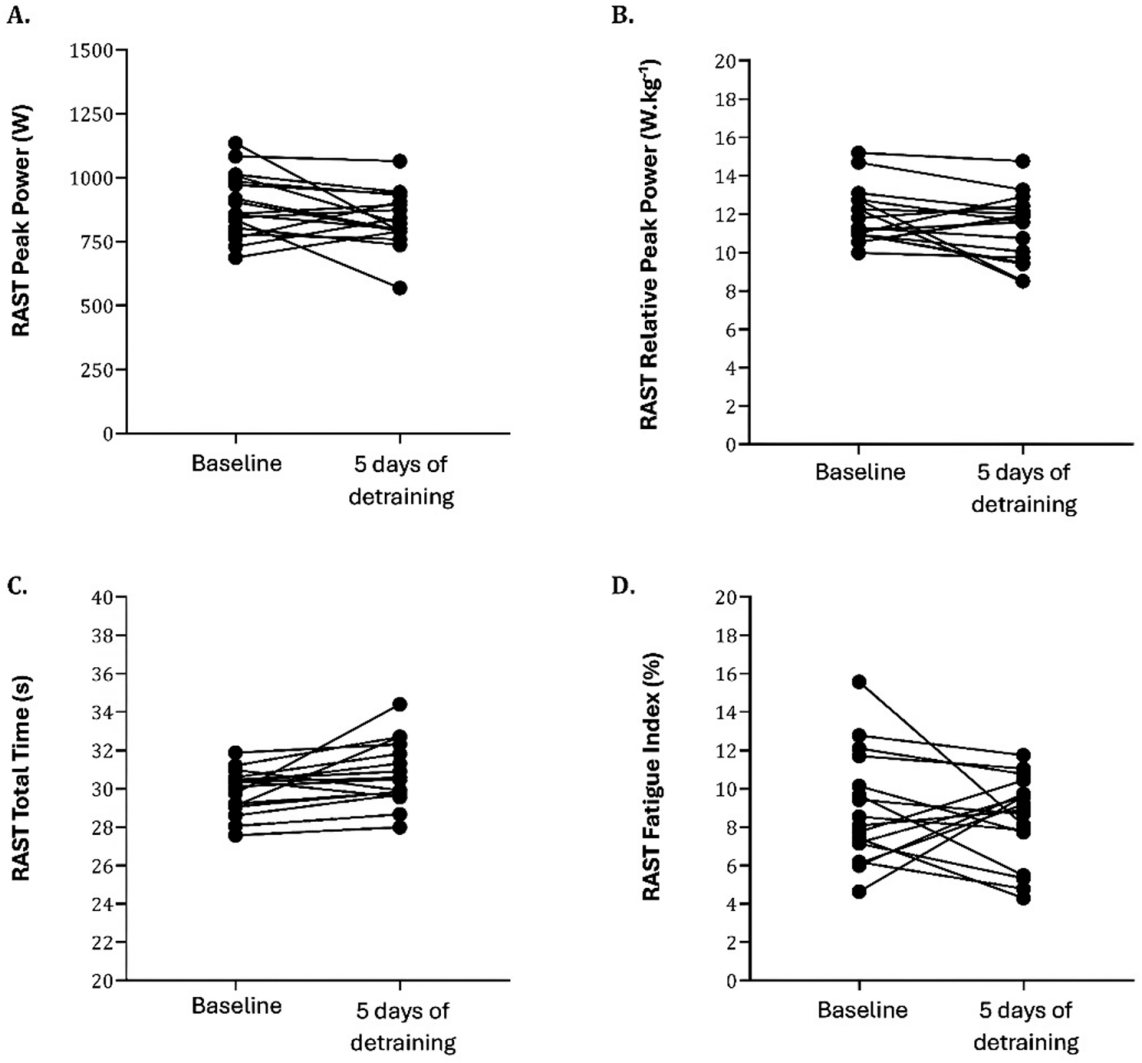
Intra-individual variation in RAST (Running Anaerobic Sprint Test). Each dot represents one player, and lines connect individual values at baseline and after 5 days of detraining. Panel **A**: Peak Power; Panel **B**: Relative Peak Power; Panel **C**: Total Time; Panel **D**: Fatigue Index

**Fig. 4:**
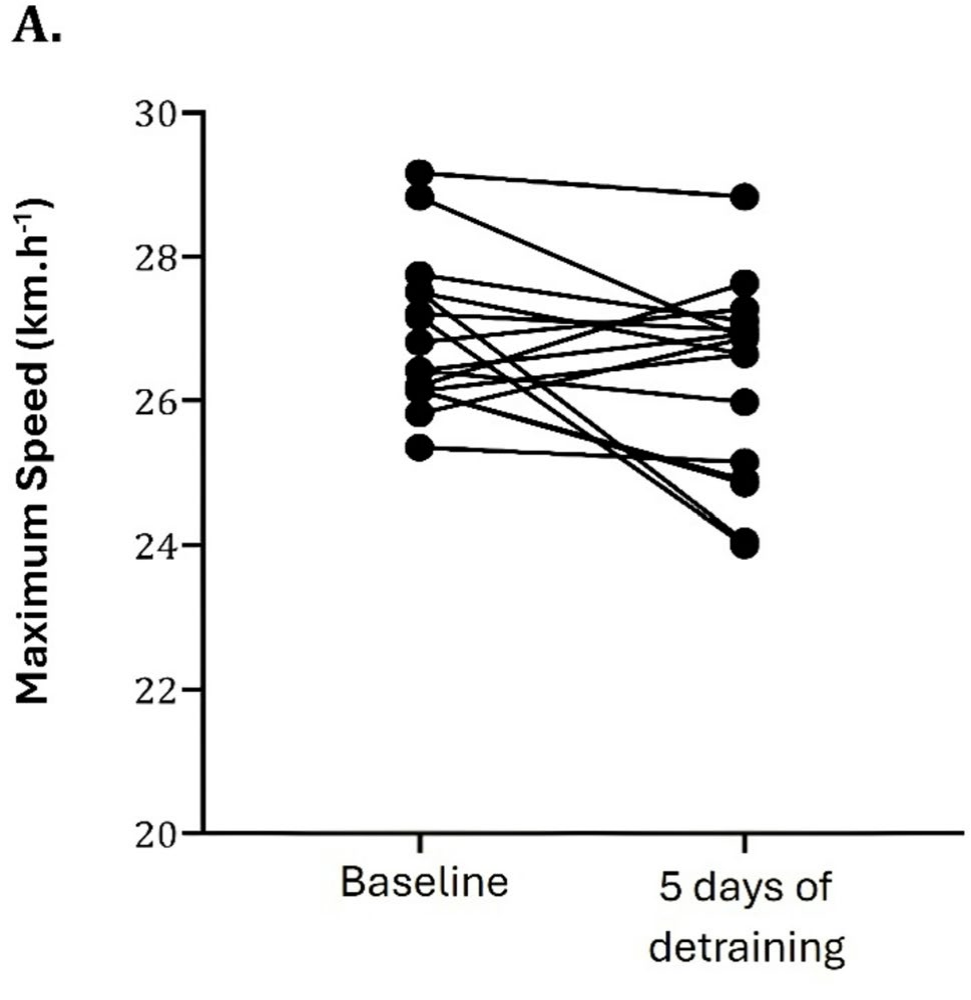
Intra-individual variation in the maximum speed of the RAST (Running Anaerobic Sprint Test). Each dot represents one player, and lines connect individual values at baseline and after 5 days of detraining

Visual data inspections were made using stem-and-leaf diagrams and box-and-whisker plots to examine central tendency and data variation. Subsequently, the Gaussian distribution of variables was confirmed with normal Q.Q. plots and the Shapiro-Wilk test. The Levene’s test was used to check the homogeneity of variance. Subsequently, descriptive statistics (mean ± standard deviation) were calculated for each moment (before the interruption and after five days of detraining). The comparison between the two-time moments was conducted using two-tailed paired t-tests. Football players’ Cohen d-values were calculated and interpreted as follows [55]: d ˂ 0.2 (trivial), 0.2 ≤ d < 0.6 (small), 0.6 ≤ d < 1.2 (moderate), 1.2 ≤ d < 2.0 (large), 2.0 ≤ d < 4.0 (very large) and d ≥ 4.0 (nearly perfect). Intraindividual differences were individually calculated, testing the effect of detraining conditions, and graphically visualised. Only the significant variables were presented in Figs. 3 and 4. Missing data were handled using listwise deletion, whereby only participants with complete pre- and post-assessment data for each variable were included in the respective analyses. Statistical significance was set at 5%. These analyses were performed using SPSS version 20.0 (SPSS et al. Company, N.Y., USA) and GraphPad Prism (version 5.00 for Windows, GraphPad Software, San Diego, California, USA, www.graphpad.com).

#### Body composition

Assessment with a bio-impedance device (InBody 770, Cerritos, CA). The measurement took place early in the morning, with the participants in a fasted state and wearing only underwear. No further pre-test restrictions were imposed. During the assessment, the participants did not speak, remained barefoot, standing with both arms 45° away from the torso, with both feet and hands in contact with the platform’s electrodes. The following variables were analysed: (1) Body mass in Kg, (2) Percentage of fat mass, (3) Fat mass in Kg, and (4) Fat-free mass in Kg.

## Results

Table 1 summarises the mean, standard deviation and sample size for body size, body composition (*n* = 21) and functional parameters (jumping (*n* = 17), abductors (*n* = 19) and adductors (*n* = 21) strength and RAST outputs (*n* = 17)) before the interruption and after five days of detraining. Table 1 also demonstrates the estimation of effect sizes for the different assessments.

**Table 1:** Descriptive Statistics, t-Test, and Effect Sizes for Body Composition and Functional Parameters Pre- and Post-Detraining

Variable	*N*	Moment 1 (Pre)		Moment 2 (Post)		Mean Difference	t-Test		Magnitude of effects
		mean	standard deviation	mean	standard deviation	(Post-Pre)	t	*p*	Cohens’d (95% CI)
Body mass, kg	21	75.08	8.45	75.36	8.33	0.28	1.538	0.070	0.336 [-0.108; 0.772]
Fat-free mass, kg	21	66.48	7.01	66.57	6.90	0.09	0.412	0.342	0.090 [-0.340; 0.517]
Fat mass, kg	21	8.60	2.46	8.78	2.56	0.18	0.979	0.170	0.214 [-0.222; 0.644]
Countermovement Jump, cm	17	41.22	4.64	41.46	5.34	0.24	0.255	0.802	0.062 [-0.415; 0.537]
Squat Jump, cm	17	40.91	4.62	41.56	5.64	0.64	1.017	0.162	0.247 [-0.240; 0.726]
Adduction right limb, kg.f	21	47.14	7.91	46.77	7.85	-0.37	0.313	0.379	-0.068 [-0.496; 0.361]
Adduction left limb, kg.f	21	48.81	9.36	47.19	8.81	-1.62	1.265	0.110	-0.276 [-0.709; 0.163]
Abduction right limb, kg.f	19	48.46	7.23	48.56	6.19	0.1	0.088	0.466	0.020 [-0.430; 0.470]
Abduction left limb, kg.f	19	48.84	5.89	50.28	6.07	1.44	1.405	0.089	0.322 [-0.144; 0.780]
RAST time, s	17	29.89	1.14	30.77	1.62	0.88	2.616	0.009	0.634 [0.103; 1.149]
RAST power, W	17	892.34	125.46	837.54	108.92	-54.80	1.762	0.049	-0.427 [− 0.919; 0.76]
Maximum speed, km.h-^1^	17	26.86	1.03	26.21	1.35	-0.65	2.017	0.030	-0.489 [-0.987; 0.22]

*kg.f* kilogram-force (force exerted by 1 kg mass under standard gravity; 1 kg.f ≈ 9.81 N; see Methods for details)

### Repeated sprinting ability (primary outcome)

After the 5-day in-season break, repeated sprinting ability worsened. RAST total time increased by approximately 1.0 s (≈ 2.9%, from 29.89 ± 1.14 s to 30.77 ± 1.62 s; *p* = 0.009, 95% CI for the mean difference [-0.17; 1.60]), with a moderate effect size (d = 0.634, 95% CI for d [0.103; 1.149]). Peak power decreased by about 55 W (≈ 6.1%, from 892.34 ± 125.46 W to 837.54 ± 108.92 W; *p* = 0.049, 95% CI for the mean difference [-120.74; 11.14]), and maximum speed declined by 0.65 km.hˉ¹ (≈ 2.4%, from 26.86 ± 1.03 km.hˉ¹ to 26.21 ± 1.35 km.hˉ¹; *p* = 0.030, 95% CI for the mean difference [-1.35; 0.03]), both measures with small effect sizes (d = -0.427, 95% CI for d [− 0.919; 0.76] and d = -0.489, 95% CI for d [-0.987; 0.22], respectively). Within-group (before vs. after five days of detraining) analysis revealed a significant difference in total time to complete RAST (panel C), peak power (panel A), as shown in Fig. 3 (*p* < 0.05) and in maximum velocity (Fig. 4). On the remaining parameters, intra-individual variation was non-significant. For RAST total time (*n* = 17), 15 players (88.2%) showed a longer completion time, and 2 (11.8%) improved; for peak power, 12 players (70.6%) decreased, and 5 (29.4%) increased; and for maximum speed, 12 players (70.6%) declined and 5 (29.4%) improved. While Figs. 3 and 4 illustrate pre–post changes for the RAST variables that reached statistical significance and were considered primary outcomes, all other secondary outcomes, including those with trivial‑to‑small effect sizes and non‑significant *p*-values, are presented numerically in Table 1 to allow complete clinical interpretation.

### Secondary outcomes

Negligible differences were found in body mass, body composition, strength of abductors, and jumping ability. Although the strength of adductors on the right and left sides decreased by 0.37 kg.f and 1.62 kg.f, respectively, these pre–post differences did not reach statistical significance.

## Discussion

The present findings indicate that this short-term stoppage was associated with a poorer ability to perform repeated high-intensity efforts, namely in total time, power and maximum speed achieved in the RAST. No significant changes were seen in the other physical qualities assessed after the break. These results suggest that the brief interruption of five days had a modest impact with moderate effects (d = 0.634) on RAST total time and small effects on RAST power and maximum velocity (d = -0.427 and d = -0.489, respectively). In practical terms, the observed increase of approximately 1.0 s in RAST total time may correspond to a meaningful decrease in repeated sprint performance during match play. Furthermore, such decrements observed in the RAST outputs could be particularly relevant given that they directly reflect the ability to sustain repeated high‑intensity running efforts, which underpins many decisive actions in competitive football. However, given the absence of a randomised control group, these findings cannot be interpreted as demonstrating a causal effect of the 5-day break per se, but rather as reflecting performance changes observed under real-world, uncontrolled in-season conditions.

### Repeated sprinting ability

Relative to these moderate-to-small effect sizes obtained in some RAST variables, the observed changes may have direct implications for players’ performance on the field, given that football, although predominantly aerobic, requires bouts of high intensity and brief duration efforts, with short rest periods, triggering an interaction between the main aerobic and anaerobic systems. Moreover, it is suggested that several critical factors of successful performance outcomes in football demand high-speed actions [8, 10, 56]. The fact that these changes emerged in repeated sprinting capacity but not in maximal strength or vertical jump height suggests that short-term detraining may preferentially affect the capacity to repeat high‑intensity efforts, while leaving more structural muscular qualities and isolated explosive actions relatively preserved over a five-day interval. Thus, the most affected parameters in RAST, such as sprinting and anaerobic power, might be prioritised in the short period return to training, as outlined in the practical implications section. However, this test (RAST), while relevant to football-specific repeated sprinting, presents ecological limitations related to reactive and multidirectional features of actual match play. During a soccer match, players typically cover around 11,000 m of total distance, including high-intensity efforts such as 900 m of high-speed running, 2472 m of high-metabolic load, as well as approximately 61 accelerations and 58 decelerations (≥ 3 m·s²) [57, 58]. Hence, these results should be interpreted with caution, as they may not fully capture the complexity of on-field performance. The RAST provides information about an individual’s anaerobic characteristics in a linear running format, and therefore it is not advisable to directly transfer inferences from it to multidirectional movements and reactive decision-making traits. Furthermore, although the FI was calculated, it is not reported given the lack of statistically significant results and its poor reliability within repeated sprint assessments [54], which further limits its interpretability. Nonetheless, from a practical standpoint, a slower RAST profile following the break may indicate a reduction in readiness for back-to-back bursts of sprints, accelerations, and transitions, even if isolated performance in terms of strength and jump appears relatively stable.

The findings of this study regarding repeated sprinting performance are in line with the evidence in the literature [26, 29, 30, 34, 35]. All of these studies revealed that a short-term period of detraining yields significant negative effects. As the RAST is a test designed to assess anaerobic capacity and power, a plausible explanation for these results may be related to adaptations induced by detraining at a cellular and molecular level. From a mechanistic, hypothesis‑generating perspective, previous research suggests that short-term detraining may be associated with a decrease in the resting phosphorylation state of the sodium-potassium pump (Na+/K+) [34], potentially increasing negative effects on fast-twitch muscle fibres and on the ability to use the ATP and phosphocreatine available, accompanied by a greater production of metabolic by-products [30]. Such alterations could theoretically compromise motor unit recruitment and synchronisation and, in turn, the maintenance of repeated high-intensity efforts [29, 30, 34]. However, these mechanistic explanations remain speculative and based on prior literature, as the present study did not directly measure cellular or molecular parameters.

### Body composition and strength

In relation to body composition, trivial increases were observed in body mass, fat-free mass and fat mass, although these changes were not statistically significant. Given the relatively small sample size and the negligible magnitude of these changes, the study may have lacked sufficient power to detect subtle but potentially meaningful alterations in body composition. In the study carried out by Joo (2016) [29], a significant increase in body mass was observed (69.4 ± 6.8 kg vs. 70.1 ± 6.9 kg, *p* = 0.03) in the subjects assessed after one week of detraining, and a systematic review and meta-analysis conducted by Clemente et al. (2021) [26] reported that, in general, there is a small and significant increase in body fat after a break from training, whether short-term or long-term. Although our study showed no significant differences, these previous data suggest that short-term detraining is often associated with detrimental effects on body composition, namely an increase in the percentage of fat mass and a decrease in lean mass. This could be related to changes in lifestyle and habits during breaks, such as changes in eating and nutritional behaviour and physical activity levels [26].

It is therefore plausible that the five days of detraining in our study were not sufficient to induce detectable changes in this sample. Another possible confounder was the fact that in our study we did not control some variables like daily physical activity, dietary and fluid intake nor sleep quality, which could influence the absence of significant body composition changes. Moreover, the combination of uncontrolled lifestyle factors and limited sample sizes increases measurement variability, further limiting statistical power and raising the likelihood of Type II errors when evaluating small changes.

Regarding maximum thigh adduction and abduction strength, no statistically significant changes were detected. Other studies, even when considering different tests to measure maximum strength, concluded that interruptions of one, two, and three weeks did not significantly affect the isokinetic concentric strength peaks of knee flexion and extension [4, 23, 29, 59]. In addition, the study by Pereira et al. (2020) [27] also found no significant changes in the 1-repetition maximum test after a 26-day break.

Taken together, the non-significant findings for body composition and maximum strength should be considered preliminary, as a short period of detraining (5 days) in well-trained soccer players may still induce subtle changes that our design was not adequately powered to detect, despite similar patterns having been reported in Joo (2018) [7] after a pause period of 2 weeks. Nevertheless, insufficient control over key variables, specifically physical activity, nutrition, and sleep, likely influenced these outcomes, particularly in terms of body composition. This uncontrolled heterogeneity in lifestyle behaviours during the break likely contributes to the marked intra-individual variability observed in RAST and maximum speed performance (Figs. 3 and 4), where some players exhibited pronounced declines whilst others maintained or improved. Such variability complicates the isolation of training cessation per se as the sole driver of the observed changes, as more active players might have partially offset detraining through unstructured physical activity, whereas complete rest could have amplified fatigue dissipation or subtle deconditioning effects. Thus, the absence of significant findings should be interpreted with caution, especially since the small and variable sample size increases the risk of type II errors [60], that is, failing to detect actual effects. While no statistically significant changes were observed for body composition and strength, the trivial to small effect sizes (e.g., Adduction left limb, d = -0.276), combined with our limited sample size, indicate we cannot rule out physiologically relevant changes. These parameters may be more resistant to a 5-day break, but a larger, controlled study is needed to confirm this. Hence, the absence of statistically significant differences should not be taken as definitive evidence of no effect. Consequently, the present study should be regarded as an observational report of performance fluctuations during a short in-season break in a professional environment, where training loads, lifestyle habits, and recovery patterns could not be experimentally manipulated or strictly standardised.

While short-term detraining does not change the distribution of muscle fibres, there is the likelihood of reducing the size of fiber cross-sectional area, which may compromise force production [7, 22]. Another studies [4, 23, 24] equally referenced that the decline in muscle strength and strength related performance appears to be a consequence of reductions in muscle fiber size, particularly on account of reduced type II muscle fiber area, mitochondrial ATP production and enzymatic activities. According to our research study results, it seems to us that the break time was not enough to produce these modifications and thereby affect the strength performance of these athletes, since the 5-day break in the present study was much shorter than the 3-week break after the first half of the season in elite football players resulted in changes in skeletal muscle morphology [61]. Notwithstanding these findings, given the small sample size and trivial-to-small effect sizes, it is plausible that the study was underpowered to detect early morphological or functional adaptations that may have occurred.

### Vertical jump performance

Findings indicated a negligible, non-significant increase in the average heights obtained in the CMJ and SJ. This pattern aligns with literature reporting improvements in jump performance after brief training cessations [27, 62], whereas longer detraining periods (e.g., 3 months) have been associated with significant reductions in jump height [63]. Therefore, although our results do not show significant changes, when considered alongside previous research findings, it is consistent with the hypothesis that while long-term detraining may detrimentally affect jumping performance, shorter periods of break might potentially favour the expression of this neuromuscular capacity in well-trained athletes.

According to Jeffreys (2005) [64] and Pereira et al. (2020) [27], this pattern may reflect the relationship between readiness and fatigue (“fitness-fatigue” paradigm), whereby short breaks might allow accumulated fatigue to dissipate, potentially enhancing neuromuscular expression. It is also suggested that greater training volumes can suppress the expression of certain neuromuscular capacities, such that temporary reductions in training load during short breaks may facilitate improvements in jumping performance [27, 33].

### Practical implications

When planning training programmes (microcycles, meso- and macro-planning), technical staff should consider these findings during short in-season breaks. Appropriate and thoughtful handling of training load variation is required to achieve optimum readiness after brief cessations from training and competition. Individualisation of loads is particularly relevant for players with higher physical conditioning, who may experience greater detraining effects [30], as supported by the marked intra-individual variability observed in RAST and maximum speed responses (Figs. 3 and 4). This heterogeneity suggests some players require additional anaerobic/speed work during return-to-training, while others may need only minimal stimulus.

Overturning detraining effects remains a priority for high-performance football coaches. Accordingly, methods such as HIIT and SSGs have become quite popular in this field, as both rely on frequent direction changes, accelerations, and decelerations, effectively inducing physiological adaptations when reintroduced post-detraining [5, 65]. HIIT enables faster adaptations than SSGs due to its higher neuromuscular strain and structured delivery, while excelling in linear running speeds, agility without the ball and anaerobic metabolism, precisely the capacities most affected in our RAST results. Conversely, SSGs offer superior sport-specific benefits for agility with the ball [5, 22, 65]. Recent studies suggest that performing HIIT sessions alone or combined with SSGs, as little as two to three times per week, over a period of four to six weeks, may be an effective minimum dose for fitness restoration [5, 22]. These complementary strengths suggest that prioritising HIIT immediately post-detraining may be particularly beneficial for restoring repeated sprint ability.

Beyond training method selection, session organisation impacts reconditioning. Arslan et al. (2021) [5] demonstrated that HIIT + SSGs sequences produced lower RPE, training loads, and greater enjoyment compared to SSGs + HIIT, despite equivalent fitness gains. Thus, implementing HIIT before SSGs may facilitate internal load management and player adherence during return-to-training phases, though exercise order should always adapt to individual/contextual factors.

### Limitations

There are some limitations associated with this study that should be acknowledged, particularly regarding sample size, absence of a control group, and lack of control over lifestyle confounders.

The relatively small and variable sample size (*n* = 17–21), combined with non-random missing data (injuries, A-team selection), may have introduced selection bias and reduced statistical power, increasing Type II error risk and limiting detection of subtle effects (e.g., body composition, strength). Taken together with the absence of an a priori power calculation, these factors reinforce the preliminary, exploratory nature of the present analyses. In brief, this limited sample size constrains the generalisation of results and hinders the statistical power to detect any subtle real effects. Additionally, the inclusion of both outfield players and goalkeepers may have increased between‑player heterogeneity in performance profiles, and this should be considered when extrapolating the present findings.

Another limitation is the absence of a randomised control group, which substantially restricts causal inference. Without a comparison group maintaining controlled activity, it is not possible to definitively distinguish detraining-related changes from natural test variability, residual fatigue, seasonal influences, circadian effects, scheduling differences across testing days and daily activity. Furthermore, the absence of familiarisation trials within the RAST protocol should also be noted as a limitation, as even experienced players may exhibit day-to-day variability in exhaustive tests. Therefore, the observed differences should be interpreted as outcomes occurring under applied, in-season field conditions that were not experimentally controlled, rather than as isolated effects of the 5-day break itself.

Additionally, potential key confounding factors, including physical activity, nutritional intake, and sleep, were not fully controlled. Although the participants were advised to refrain from self-administered vigorous exercise, their activity levels were not strictly monitored, and diet, fluid intake, and sleep quality were not measured, potentially masking or amplifying detraining effects. Moreover, assessors were not blinded, which may have introduced subtle measurement or expectation bias during testing.

Lastly, the competitive level of the sample, comprising full-time players mainly competing in the 4th division, may limit generalisability to female players, youth, or older athletes across distinct competitive levels, who may display different training histories, match demands, and detraining responses.

### Future directions

Future research should prioritise expanding sample sizes and the inclusion of a randomised control group to strengthen causal inferences, better isolate the effects of detraining from external factors and improve generalisability. Implementing objective assessment tools, including activity trackers, dietary logs, and sleep monitoring devices, together with subjective measures such as perceived wellness, fatigue, and motivation, would enhance accuracy, reduce within-group variability, and help clarify the real impact of short-term detraining periods by providing a more holistic view of players’ responses. Longitudinal designs across multiple in-season breaks, incorporating biomechanical or neuromuscular metrics, could further elucidate how short-term detraining influences performance over time. Furthermore, future studies should investigate individual response variability (as visualised in Figs. 3 and 4). Identifying player characteristics, such as baseline fitness and training age, that may predict susceptibility to detraining would enable more personalised and proactive reconditioning strategies during the return-to-training phase. Overall, these directions aim to advance understanding of short-term in-season detraining and underpin the development of evidence-based strategies to mitigate its potential detrimental impact on physical condition.

## Conclusion

This preliminary observational study shows that, over the course of the season, a 5-day in-season break is associated with a significantly lower repeated sprinting ability in football players when physical performance is evaluated under real-world, uncontrolled conditions. In contrast, muscular power, assessed by performing vertical jumps (CMJ and SJ), showed minor increases without statistical significance. As for the expression of maximum thigh adduction and abduction strength and body composition, there were no significant changes after the short break from training and competition. These results emphasise that the effects of short-term detraining are nuanced, with an observed decline in repeated sprinting performance, while strength, jumping ability, and body composition did not show statistically significant changes and only trivial-to-small effect sizes. However, given the limited sample size and absence of a control group, these findings should not be interpreted as definitive evidence of a causal effect of the break itself or of the absence of changes in these parameters.

Therefore, professionals involved in planning and periodising training should be aware of these observed changes to establish priorities and devise more effective training methods and strategies to implement after the short break. During the competitive season, we know that time is limited and congested to properly develop all the players’ physical, technical and tactical abilities, which is why proper management of this process is vital.

Thus, according to previous studies, regarding different training methods to reestablish the physical fitness desired on the pitch [5, 22], as well as to our findings, practitioners may consider prioritising HIIT immediately after short breaks to efficiently address the observed reductions in anaerobic power.

Considering the preliminary nature of our findings due to small sample size, absence of a control group, and potential uncontrolled confounders, further studies are needed to examine the relationship between exercise performance and the cellular and molecular responses derived from detraining. Furthermore, we highlight the potential benefits of (1) increasing the sample size and/or (2) extending the period of detraining up to 10–14 days, in future research to (3) enhance precision and statistical power for detecting smaller effects, (4) analyse possible delayed effects and whether the inclination toward jump improvements become significant, and (5) of completely controlling external factors, such as activity levels, during the detraining period. It is essential to understand whether short phases of detraining tend to be beneficial or harmful for soccer players, so that more coherent and consistent conclusions can be drawn on this subject.

## Supplementary Information

Supplementary Material 1.

## Data Availability

All relevant data are within the manuscript and its supplementary information file.
